# 分子印迹技术在疾病诊断和治疗领域的研究进展

**DOI:** 10.3724/SP.J.1123.2025.06036

**Published:** 2026-01-08

**Authors:** Ran ZHU, Ganping CAI, Haijiao ZHENG, Qiong JIA

**Affiliations:** 吉林大学化学学院，吉林 长春 130012; College of Chemistry，Jilin University，Changchun 130012，China

**Keywords:** 分子印迹技术, 分子印迹聚合物, 疾病生物标志物, 诊断, 治疗, molecular imprinting technology （MIT）, molecularly imprinted polymers （MIPs）, disease biomarkers, diagnostics, therapeutics

## Abstract

疾病生物标志物在疾病早期诊断、准确分型、预后评估及靶向治疗等方面发挥着重要的作用。抗体和适配体等生物识别元件虽具备高特异性，但它们生产成本高，在复杂基质中稳定性低，批次间差异性大，难以满足对高通量、低成本及多场景应用的需求。基于分子印迹技术（MIT）制备的分子印迹聚合物（MIPs）具有成本低、选择性高、物理化学性质稳定等优势。近年来，MIT凭借其灵活的定制化设计功能，不仅适用于分析不同类型的疾病生物标志物，而且能够整合信号转导或刺激响应等功能，以满足多样化应用场景需求，因此在疾病诊断和治疗中受到广泛的关注。本文系统综述了近年来MIT在疾病诊断和治疗领域的研究进展，主要聚焦于不同类型疾病生物标志物的印迹技术及其相关应用研究，并分析了当前面临的挑战与未来发展方向。

## Abstract (en)

Disease biomarkers play important roles in modern medicine， enabling early disease diagnosis， precise subtyping， prognostic evaluation， and targeted therapy. Conventional biorecognition elements， such as antibodies and aptamers， offer high specificity but suffer from inherent limitations， including high production costs， instability in complex biological matrices， and significant batch-to-batch variation. Collectively， these constraints restrict their scalability and versatility for high-throughput， cost-effective， and multi-scenario clinical applications. Molecularly imprinted polymers （MIPs）， prepared via molecular imprinting technology （MIT）， have emerged as highly robust and promising synthetic receptors. During their formation， functional monomers assemble around a target molecule， and highly specific binding sites are formed after polymerization and template removal. MIPs exhibit a unique combination of advantages， ranging from cost-effectiveness to high selectivity， as well as high affinity comparable to natural counterparts， and outstanding physicochemical stability under harsh conditions. Driven by their highly flexible and customizable design capabilities， MIP development has witnessed remarkable advancements in recent years. These polymers are suitable for analyzing different types of disease biomarkers， ranging from proteins， peptides， and saccharides to complex biological entities such as whole cells and extracellular vesicles. Moreover， MIPs can be functionally engineered to integrate signal transduction or stimuli-responsive features， facilitating the creation of intelligent biomedical platforms. This review systematically summarizes the progress in applying MIT for disease diagnostics and therapeutics. It first elaborates on diverse imprinting techniques specifically tailored for different biomarker classes， including proteins， peptides， saccharides， cells， and extracellular vesicles. Subsequently， the article provides a comprehensive overview of their applications in diagnostics， encompassing biosensing， bioimaging， and bioseparation， as well as therapeutic applications， including drug delivery， photothermal and photodynamic therapy， biotoxin removal， and cell behavior regulation. Additionally， critical challenges hindering clinical translation are discussed， such as biocompatibility， long-term toxicity， high large-scale manufacturing costs， and the lack of standardized clinical validation protocols. Finally， promising future directions are outlined， emphasizing the development of biodegradable materials， integration with artificial intelligence， and adoption of green synthesis strategies. These synergistic approaches are expected to stimulate innovation， enabling the safe， reliable， and scalable translation of MIPs into real-world biomedical and clinical applications. In summary， MIPs represent a versatile， robust， and economically viable alternative to conventional biorecognition elements. Their customizable nature， combined with functional engineering capabilities， positions MIPs as a promising technology in disease diagnosis and therapy. Future efforts focusing on clinical translation， sustainability， and intelligent platform integration are likely to accelerate their adoption across diverse biomedical fields.

疾病生物标志物是指体内发现的具有医学或生物学意义的特定物质，包括小分子化合物、肽、蛋白质和糖类等^［1］^。它们与各种疾病状态密切相关，能够直接或间接地反映疾病的发生或发展状况^［2］^。随着全球人口老龄化的加剧和疾病谱系的日益复杂，疾病诊断和治疗需求正朝着精准化、动态化、个性化方向发展^［3］^。在此背景下，疾病生物标志物在疾病诊断和治疗领域的重要性日益凸显，不仅能够为早期诊断、准确分型和预后评估提供关键依据，还可作为生物成像等靶向诊断和治疗技术的理想目标物。此外，研究者们还通过代谢组学、蛋白质组学等前沿组学技术，不断发现和验证新型标志物。

尽管抗体、适配体等针对疾病生物标志物的生物识别材料具有优异的特异性，但其生产成本高昂，易受温度、pH等环境因素干扰，且批次间稳定性较差^［4，5］^。分子印迹技术（molecular imprinting technology，MIT）提供了一种替代方案，它通过模拟抗原-抗体识别机制，制备具有高亲和力的分子印迹聚合物（molecularly imprinted polymers， MIPs）。MIPs的制备过程通常包含3个步骤：首先，模板分子与功能单体基于分子间作用力通过预组装而形成模板-单体复合物；随后，在交联剂存在下，该复合物发生聚合反应，使单体围绕模板固化形成刚性网络；最后，通过物理或化学方法移除模板分子，在聚合物基质中留下在空间形状和功能基团排列上与模板高度匹配的特异性识别位点^［6-8］^。MIPs的模板选择范围广泛，小至有机分子，大至蛋白质或整个细胞，研究者们能够根据目标分析物的特性，“量身定制”具备高选择性的识别材料。更重要的是，将MIPs作为识别元件，与传感、成像、质谱分析以及药物释放等技术联用^［6，9-12］^，在应对复杂疾病方面展现出良好的应用潜力。

MIPs凭借其卓越的分子识别能力与高度可定制的设计灵活性，已成为疾病诊断和治疗领域极具前景的高特异性材料。其在生物传感、生物成像、生物分离、药物递送、光热和光动力疗法、清除生物毒素、调控细胞行为等多种应用中展现出优异的性能，应用场景覆盖了从高灵敏度疾病早期诊断到靶向治疗。本文系统性地综述了针对不同类别疾病生物标志物的MIPs前沿技术，介绍了MIPs的设计合成原理，并总结了这些技术在疾病诊断和治疗领域取得的最新研究进展。最后，总结了当前制约MIT广泛应用的主要挑战。并据此展望了其未来的发展方向。

## 1 分子印迹技术

### 1.1 蛋白质印迹技术

蛋白质是由氨基酸经肽键连接形成的大分子，具有特定的三维空间结构，是生命活动的主要执行者。其功能异常与多种疾病的发生发展密切相关^［5，7］^。然而，蛋白质由于自身的分子质量大、复杂结构及其固有的三维构象，易受聚合条件（如溶剂极性、聚合温度）影响而发生不可逆的改变，使完整蛋白质印迹技术面临模板去除难和印迹效果不佳的挑战^［8，13］^。

表面印迹技术将模板蛋白质先固定在载体材料表面，再引发聚合，该方法有效改善了传质效率和模板可及性^［7］^。然而，光聚合或原子转移自由基聚合等聚合方法通常存在由紫外线引起的模板蛋白质构象变化或残留金属的潜在毒性等问题^［12］^。此外，为了避免MIPs的团聚和反应分散体系的凝胶化，通常只能在低单体浓度下进行聚合，导致模板与单体之间的预组装不充分，从而使得MIPs的识别位点数量少，亲和力较弱。Zhang课题组^［14］^最近开发了一种表面酶介导聚合与智能肽交联剂结合的印迹策略。利用自由基在载体-溶液界面处产生的原理和肽交联剂pH响应的构象转换，解决了完整蛋白质印迹效率低和模板去除困难的问题。具体而言，该MIPs的合成采用温和的水体系，聚合由预先固定在载体表面的葡萄糖氧化酶引发，因此聚合只发生在载体表面，在单体含量达40 mg/mL时仍保持MIPs的单分散性（聚集度指数，PDI<0.1），避免了聚合过程中MIPs的团聚和高浓度单体聚合时产生的凝胶化。此外，作者设计合成的pH响应型肽交联剂在pH 5.0时形成*α*-螺旋构象固定模板，而在pH 7.4时转为卷曲构象，无需强极性溶剂即可温和释放模板。该策略成功实现了对牛血清白蛋白的高效印迹，其印迹因子达到了10.36^［15］^。

基于完整蛋白质模板的MIPs制备普遍采用温和反应体系，这与蛋白质构象敏感性的本质特性密切相关^［7，16，17］^。皮克林乳液聚合是一种采用温和条件制备MIPs的新方法。相较于常规乳液聚合技术中使用易使蛋白质变性的表面活性剂用作乳化剂的方法，皮克林乳液聚合采用固体纳米颗粒替代表面活性剂，使固体颗粒在油/水界面发生不可逆吸附，形成物理屏障，从而解决了表面活性剂的毒性问题^［12，18］^。例如，Sun等^［19］^使用表面改性的金属有机框架作为乳化剂，结合表面印迹技术，合成了一种特异性识别牛血红蛋白的MIPs，并将其成功用于牛血红蛋白的特异性吸附与分离。

### 1.2 肽印迹技术

相较于完整蛋白质模板，蛋白质特征肽段常被用作蛋白质印迹的替代模板。通过精准捕获蛋白质的功能性肽段，从而识别整个靶蛋白。该技术既降低了全长蛋白质的制备成本，又能有效规避完整蛋白质在印迹过程中因构象变化导致的识别效率损失问题。据此，表位印迹策略应运而生，在表位分子印迹技术（EMIT）中，表位模板需要是靶蛋白的部分结构或片段，对于暴露可及C端或N端的蛋白质，可使用其末端肽段作为模板进行印迹^［8］^。然而，并非所有蛋白质都具备这类易于印迹的线性末端。为了突破这一局限，研究者们开发了构象表位印迹策略^［20，21］^。该策略将目标表位的三维空间构象纳入识别因素，显著提升了识别的特异性。

在用于识别蛋白质的肽印迹技术中，固相合成法由于其高生物相容性和高选择性，展现出显著的应用潜力^［22］^。该印迹技术先将肽共价固定在固相载体表面，随后在模板周围进行聚合，所得产物经溶剂洗涤以去除未反应的单体和低亲和力聚合物。该方法应用具有温度敏感性质的*N*-异丙基丙烯酰胺，借助其温度响应特性，通过升温实现高亲和力MIPs的洗脱。（图1）。这种方法的主要优势在于模板分子被锚定，利于模板分子的精确排列产生更均一的识别位点，从而显著提升亲和力。同时，省去了烦琐的模板洗脱步骤，简化了后续的分离纯化过程，实现了模板重复利用并易于自动化操作。此外，固相合成法还可以在聚合中通过引入荧光物质^［23］^、电活性基团^［24］^、磁性组分^［25］^，赋予MIPs多功能性。最近，基于固相合成法开发的双模板肽印迹技术，能够在印迹靶向表位模板的同时实现药物模板的负载，在疾病与治疗领域展现出广阔的应用前景^［26］^。

**图1:**
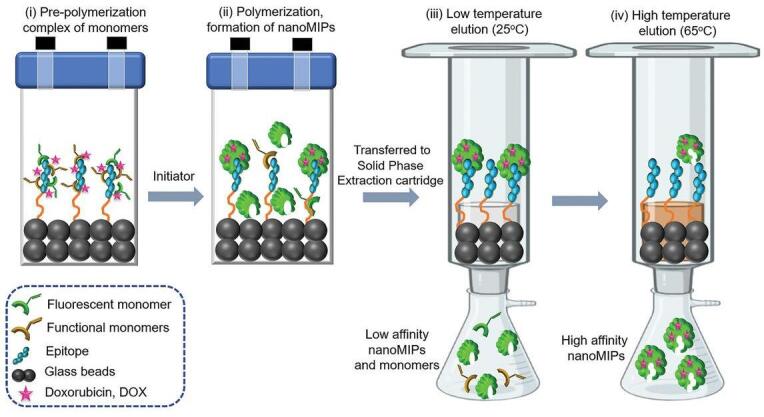
固相合成法示意图^［26］^

另外，有些肽段（如胰岛素C肽）可以直接反映特定病理状态，已被报道用作重要的疾病生物标志物。Liu团队^［27］^开发了一种分子印迹及包覆策略，该策略通过在MIPs表面制备惰性薄层包衣，显著降低了MIPs在识别过程中的非特异性吸附。利用该策略成功制备了用于检测人尿中C肽的MIPs，为糖尿病诊断提供了有效的方法。此外，在蛋白质组学研究中，对特定翻译后修饰肽段（如磷酸化肽、糖基化肽）的选择性富集与分析也引起了广泛关注。Liu课题组^［28］^开发了基于磷酸化肽印迹的MIPs，该材料表现出优异的特异性识别磷酸化肽的能力，并成功应用于复杂生物样本中低丰度磷酸化肽的高选择性富集及后续质谱分析。Hao等^［29］^通过引入印迹后修饰策略，提升了对*N*-肉豆蔻酰化肽的鉴定效果。该研究通过共价印迹结合醛基转化反应在印迹空腔内引入羟基和羧基，显著提升了对肉豆蔻酰化肽的选择性与吸附容量。将该分子印迹整体柱与固定化酶反应器集成于微流控芯片，实现了MCF-7细胞蛋白质提取物的在线变性、酶解及富集一体化流程，7 h内成功鉴定出1 296种蛋白质和5 670种肽段，其中包括71种肉豆蔻酰化蛋白和78种肉豆蔻酰化肽，较传统方法效率提升3倍以上，为复杂生物样本中蛋白质翻译后修饰研究提供了高效分析平台。

### 1.3 糖类印迹技术

糖类（包括单糖、寡糖和聚糖）广泛参与细胞黏附、信号传导及免疫防御等关键生理过程，并与病原体感染、炎症反应和肿瘤发展等病理机制密切相关^［4］^。蛋白质的异常糖基化及细胞表面糖结构的改变，常与疾病发生发展直接关联，使糖类成为重要的疾病生物标志物和潜在治疗靶点。然而，糖类固有的弱免疫原性及结构高度相似性，导致传统识别配体（如抗体和凝集素）在亲和力、选择性、稳定性方面存在显著局限，并阻碍其临床应用。MIPs凭借其特异性，为突破这一瓶颈提供了新路径。

硼酸亲和可控定向表面印迹策略（图2）是一种基于硼酸亲和与和表位定向的分子印迹技术^［30］^。在碱性条件下，硼酸基团与顺式二醇形成稳定的五元或六元环酯共价键；而在酸性条件下，这种结合被破坏，从而实现模板分子的温和去除。该策略通过精准调控模板分子的取向及印迹层厚度，在聚合物表面构建与目标糖分子三维结构高度匹配的空腔，显著提升了MIPs的识别性能。另外，适配体-MIPs杂化策略^［31］^将具有高特异性和亲和力的核酸适配体引入MIPs体系，使其作为功能单体直接参与印迹过程。在不改变材料尺寸、形貌和分散性等物理性质的前提下，大幅增强了MIPs的亲和力。上述策略通过协同硼酸亲和与生物识别机制，实现了超越单一配体体系的识别性能，提升了对结构相似糖分子的精准区分能力。

**图2:**
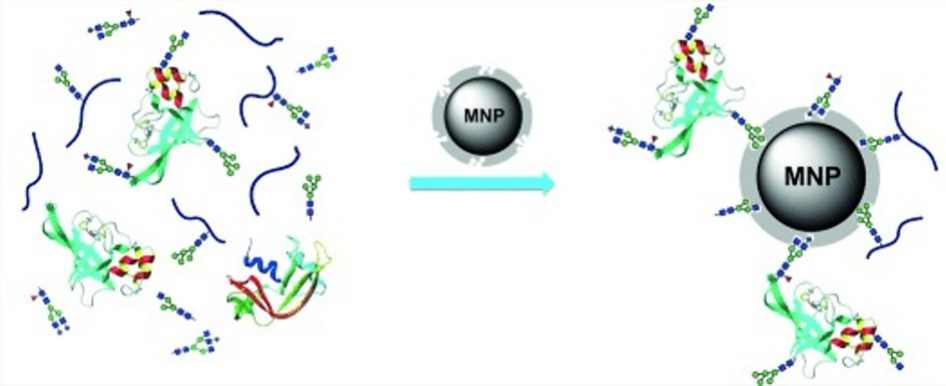
硼酸亲和聚糖定向表面印迹^［30］^

### 1.4 细胞印迹技术

随着MIT在生物识别领域的持续发展，细胞识别也受到越来越多的关注^［32］^。然而，细胞固有的结构复杂性、微米级尺寸、环境敏感性与膜流动性，给印迹技术带来了严峻挑战^［33］^。

用于细胞识别的MIPs印迹技术可分为两类：全细胞印迹和表面成分印迹。全细胞印迹以完整细胞为模板，通过精准复刻细胞形态学特征实现物理匹配，同时利用印迹空腔表面化学基团与脂质、蛋白质等细胞膜组分的特异性相互作用增强识别能力^［32］^。该技术在细菌识别中成效显著，因其形态刚性，可采用光刻^［34］^、微接触冲压^［35］^、皮克林乳液聚合^［36］^或胶体印迹^［37］^等技术直接制备印迹层。然而，哺乳动物细胞的动态膜结构易导致表面形变，使形态识别可靠性降低；且印迹生成的微米级载体易在体内循环中被截留，从而限制其生物递送应用。

表面成分印迹是对细胞表面的蛋白质和糖类等独特表位进行印迹。考虑到细胞膜分子的方向，研究可通过定向固定模板匹配细胞膜上分子的取向，提高对细胞识别的靶向性。另外，可以通过引入聚（2-甲基丙烯酸羟乙酯）和聚乙二醇等聚合物进行表面修饰，减少非特异性吸附和提升MIPs的生物相容性^［38，39］^。在实际应用中，细胞印迹技术已用于诊断传染病^［40］^及选择性识别细胞和调控细胞行为，如循环肿瘤细胞的高活性捕获与释放^［41，42］^及抗肿瘤治疗^［43-45］^等。

### 1.5 细胞外囊泡印迹技术

细胞外囊泡（extracellular vesicles， EVs）是由细胞释放的脂质双分子层包被颗粒，主要包括外泌体、微囊泡和凋亡小体等亚型^［46］^。其内部富含蛋白质、核酸、脂质等多种生物活性分子，广泛存在于各种体液中，可作为无创液体活检的标志物，在疾病诊断领域具有重要的价值^［47］^。然而，EVs在尺寸和分子组成上存在高度异质性，传统分离方法（如超速离心）所得到的样本易混入杂质，且可能导致EVs聚集或结构损伤^［48］^，难以满足分子印迹对高纯度模板的需求。

针对上述EVs印迹中高纯度模板获取方面的挑战，Takeuchi等^［49］^采用二氧化硅纳米颗粒（SiO_2_ NPs）作为EVs尺寸模拟的虚拟模板，结合印迹后修饰技术，实现了泪液样本中EVs的选择性分离与原位分析，有效解决了EVs获取的难题。Liao等^［50］^则直接以患者血清中完整EVs作为“模糊模板”制备MIPs，联合适配体介导的聚集发光信号系统，实现对未知表面蛋白EVs的高灵敏检测。

除此之外，研究者们还发展了表位印迹策略，通过靶向EVs表面特征性糖蛋白，实现了对其特定亚型的特异性检测。例如，Luo等^［51］^报道了一种基于CD63特征性三糖结构识别的电化学传感器，该策略能够特异性地捕获和检测CD63阳性sEVs亚群，实现了对医疗废水和尿液中目标亚群的灵敏、便捷、低成本分析，这对于预防传染病传播和监控医疗废物污染具有重要价值。

采用表位印迹策略虽不需要高纯度EVs作为印迹，但EVs表面成分的复杂性使得该策略过度依赖特定表位，可能导致非典型EVs亚群的漏检^［46，47，50］^。针对EVs表面成分的异质性带来的漏检问题，Chen等^［52］^提出磷脂分子定向印迹策略，即以TiO_2_作为纳米核精准锚定EVs的磷脂磷酸基团，通过反相微乳液技术，分别以磷脂酰丝氨酸、鞘磷脂等4种关键磷脂为模板制备MIPs。该策略可以成功捕获肝癌患者尿液EVs，而且这种多模板分别印迹的策略能够抵消EVs的异质性对检测效果的影响。Yin等^［53］^以完整EVs作为印迹模板制备MIPs，开发了集成式水凝胶平台。当MIPs特异性捕获EVs后，利用表面增强拉曼散射（SERS）纳米标签同步标记EVs表面的4种蛋白质（CD63/EpCAM/MUC1/PDL1），并结合机器学习算法构建泌尿肿瘤早期筛查方案。这些策略通过多模板设计和检测技术整合，有效克服了异质性干扰，推动了EVs在疾病诊断中的应用。

## 2 MIT在疾病诊断和治疗领域的应用

### 2.1 疾病诊断

#### 2.1.1 生物传感

MIPs作为特异性识别元件，兼具高选择性识别能力、优异的稳定性及多传感技术兼容性（电化学/光学），可用于构建生物传感平台并实现疾病生物标志物的高灵敏检测。这种“特异性识别-传感技术”的联用，具备高选择性、高灵敏度、实时检测能力，在疾病诊断中发挥着重要的作用。

MIT凭借其稳定性高及通用性强等优势，特别适合开发操作简便、响应快速的即时检测生物传感平台，对实现疾病的现场筛查具有重要价值。Zhou等^［54］^开发了一种便携式智能手机集成荧光传感器（Si-CD/g-CdTe@MIP），用于克罗恩病的早期诊断。该传感器采用气体喷涂技术，将硅烷化碳点与CdTe量子点复合的MIP均匀涂覆在毛细管内壁，实现了对关键生物标志物溶菌酶（LYZ）和铁离子（Fe³⁺）的高灵敏同步检测。该传感器通过单波长激发（360 nm）产生双荧光信号：Fe^3+^选择性猝灭蓝光，LYZ增强绿光。结合智能手机色彩分析应用软件（APP），将荧光颜色转化为绿红通道比值（G/R），实现现场可视化检测，检出限为0.32 nmol/L（LYZ）和0.65 nmol/L（Fe^3+^）。该传感器具备抗干扰强、稳定性好（30天）、操作简便等优势，为克罗恩病诊断提供了实用的即时检测工具。Li等^［55］^报道了一种基于还原氧化石墨烯/聚多巴胺分子印迹聚合物（rGO/PDA-MIP）的慢性肾病即时检测平台。该技术可同步检测肌酐、尿素、人血清白蛋白3种关键生物标志物。通过多巴胺在氧化石墨烯（GO）表面聚合形成印迹层，同时使部分GO还原为rGO，提升了材料的导电性与比表面积。该平台集成多通道电化学系统，利用差分脉冲伏安法实现了多重检测，具备fmol级检出限及宽检测范围。

MIPs用于生物传感的优势不仅体现在即时检测平台的设计上，其强大的分子识别能力更使其能适配蛋白质、聚糖等结构多样的关键疾病生物标志物，进而构建高灵敏度、高选择性的检测系统。本课题组^［56］^以神经元特异性烯醇化酶（NSE）的C端和N端表位作为印迹模板，分别在玻璃板和银纳米颗粒上制备了两种分子印迹聚合物（hg-MIP），构建了用于小细胞肺癌诊断的SERS传感平台。其中，表位与载体间的固定基于*β*-环糊精和偶氮苯之间的主客体相互作用，利用偶氮苯在可见光/紫外光下的光响应特性，实现模板的结合和洗脱。C端印迹的玻璃板捕获NSE后，N端印迹的银纳米颗粒作为二次识别元件特异性识别被捕获到的NSE，实现人血清中NSE的高灵敏检测，其检出限低至0.01 ng/mL。Liu课题组^［57］^报道了一种双模态比例免疫测定法，如图3所示，3个MIPs正交识别甲胎蛋白（AFP）的N端表位、C端表位和AFP上的聚糖，特异性捕获糖蛋白后通过SERS和激光解吸电离质谱检测技术获得两个独立的聚糖和糖蛋白的比值，用于肝细胞癌的精确诊断。

**图3:**
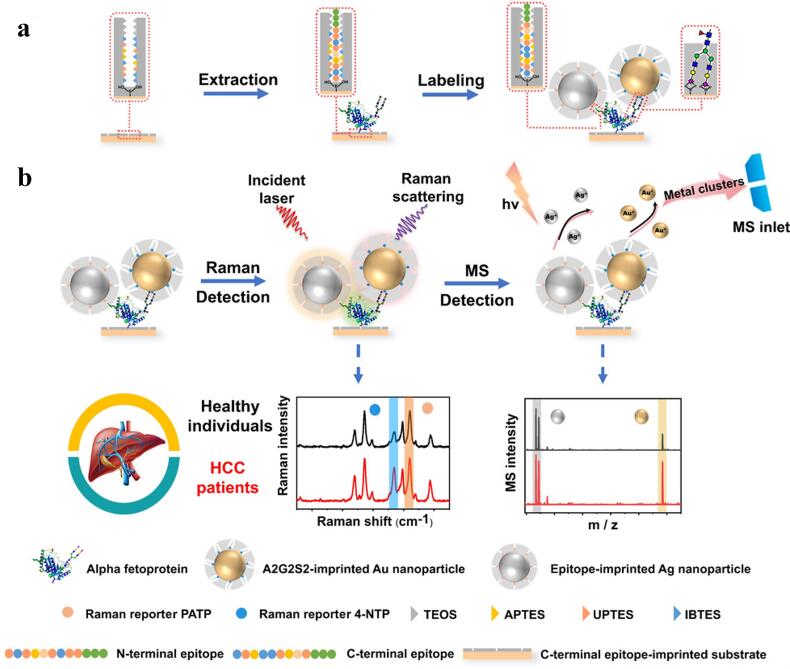
分子印迹聚合物在生物传感中的应用^［57］^

#### 2.1.2 生物成像

生物成像利用光谱或波谱等技术直接获得生物组织和细胞的微观结构及分子分布图像，通过清晰显示组织器官病变或肿瘤位置等，为疾病早期诊断提供关键信息。实现这种精准定位的核心在于高特异性的靶向识别。

肿瘤细胞表面常存在糖基化水平改变及特定蛋白质受体过表达等特异性疾病生物标志物。MIPs能够特异性识别这些标志物，从而靶向肿瘤细胞。此外，相较于抗体等生物识别分子，MIPs不仅制备相对简单，还具有优异的稳定性，不易被蛋白酶和核酸酶降解^［10］^。例如，Zhang等^［58］^以前列腺特异性膜抗原（PSMA）表位为模板，通过掺杂荧光单体，制备了一种荧光标记纳米凝胶，能够靶向识别PSMA过表达的前列腺癌细胞并实现生物成像，从而区分正常细胞与癌细胞。该材料在实现高特异性识别的同时，兼具良好的生理稳定性和低细胞毒性，为前列腺癌诊断提供了有效解决方案。

量子点（QDs）和上转换纳米颗粒（UCNPs）等纳米粒子作为生物成像信号源，能有效克服有机小分子染料荧光易猝灭的问题。然而，这些纳米粒子通常需要表面修饰识别配体以实现靶向识别，而配体偶联易引发非特异性吸附，导致特异性降低；此外，一些油相合成的纳米材料还面临着功能化困难的挑战。针对这些问题，Liu课题组^［59］^提出了一种通用性强的反相微乳液表位定向表面印迹及包覆方法（ROSIC）。如图4所示，该方法通过在纳米粒子（如QDs、Fe_3_O_4_ NPs、Ag NPs、UCNPs）表面构建具有精确识别位点的分子印迹薄层，并结合包覆策略，有效抑制了非特异性结合，实现了高亲和力和高特异性的靶向识别。基于此方法，该研究以QDs为核心，针对人表皮生长因子受体-2（HER2）和跨膜糖蛋白非转移基因B（GPNMB）2种典型癌症生物标志物，成功开发了荧光纳米探针，并在三阴性乳腺癌（TNBC）细胞及小鼠体内实现了靶向荧光成像。

**图4:**
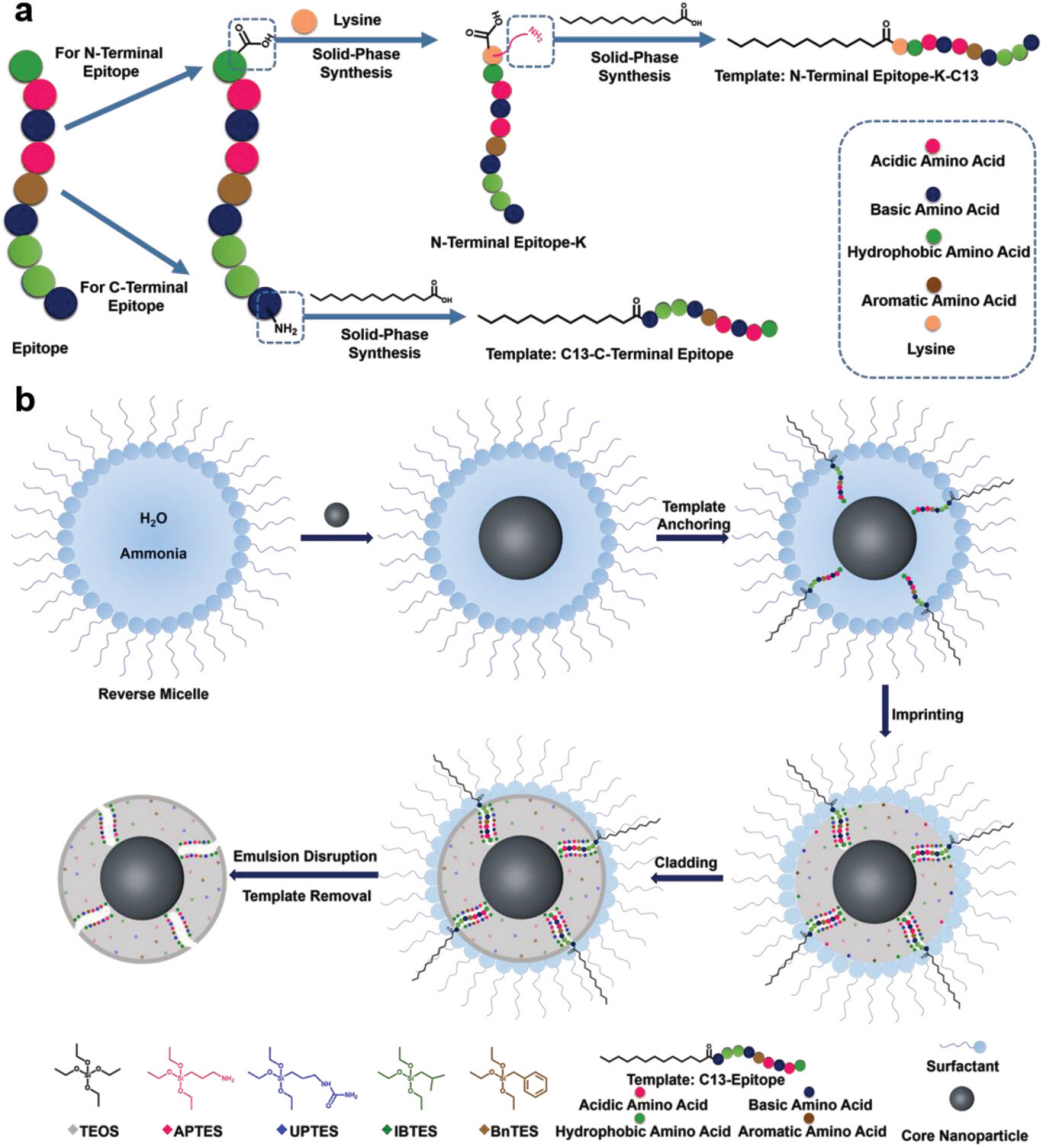
分子印迹聚合物在生物成像中的应用^［59］^

MIPs的通用性还为多模态成像提供了可能。Ren等^［60］^以Tn抗原的糖基表位（Gal-NAc）为模板，基于钆（Gd）掺杂的荧光硅纳米颗粒（SiNPs）开发了一种双模态成像探针。该探针能特异性靶向Tn抗原过表达的癌细胞，结合了荧光成像的高灵敏度和磁共振成像（MRI）的高空间分辨率优势，有效克服了单一荧光成像的组织穿透性局限。

#### 2.1.3 生物分离

生物分离作为从复杂生物样本中高选择性分离纯化特定目标物质的关键技术过程，是后续检测与分析的基础。循环肿瘤细胞（CTCs）是癌症等疾病的早期诊断和预后评估的关键信息来源，但其在血液中的含量极低。因此，高效分离纯化CTCs对于获取可靠的诊断样本至关重要。Sun等^［42］^设计了一种靶向CTCs表面唾液酸化聚糖的双组氨酸配体，并将该配体与细胞印迹技术相结合，构建了具有良好生物相容性和优异抗干扰能力的水凝胶材料。这种材料的印迹空腔能与细胞形状精确匹配，从而在复杂血液环境中实现>95%的CTCs捕获效率，并可通过弱碱性溶液无损释放活细胞。此外，运用机器学习算法，将CTCs与肝癌的其他诊断指标（AFP和自然杀伤细胞）联用，结果表明其对肝癌诊断的准确率高达94%。此外，该材料还展现出优异的可重复性（可重复使用10次）和经济性（每个样本6.68美元），为肝癌早期筛查提供了一个高性价比的解决方案。

EVs携带丰富的蛋白质、脂质、代谢物等生物分子信息，是潜在的疾病生物标志物来源。然而，体液样本中的EVs与大量游离蛋白、脂蛋白等共存，其高效、高纯度富集是后续精准组学分析的前提条件。Lu等^［61］^基于磷脂酰丝氨酸分子印迹聚合物（PS-MIPs）开发了一种高效EVs富集技术，用于多种体液中EVs的代谢组学分析。通过磁性PS-MIPs可从血浆、尿液、羊水、脑脊液、唾液5种体液中快速分离EVs，结合高分辨质谱技术，首次系统绘制了体液及其EVs的代谢图谱。研究发现，EVs含有大量体液来源特异性代谢物（仅29.2%与体液共有），且不同来源EVs代谢谱差异显著。作者进一步以肝癌血浆样本为模型，鉴定出7种潜在疾病生物标志物。相较于传统的超速离心法，该方法的捕获效率更高。这些技术的发展显著提升了从复杂生物样本中获取关键信息的能力，有助于开发更可靠的诊断工具。

### 2.2 疾病治疗

#### 2.2.1 药物递送

药物递送系统在疾病治疗领域，特别是恶性肿瘤等重大疾病的治疗中具有重要的价值。通过将化疗药物精准递送至肿瘤组织，可降低对正常组织的毒副作用并提高疗效。MIPs通过构建高密度且高亲和力的特异性载药位点，能够提升载药量并实现长效缓释。Bărăian等^［62］^开发了一种基于钙交联海藻酸钠-聚（*N*-异丙基丙烯酰胺）（ALG-PNIPAm）接枝共聚物的分子印迹水凝胶，用于递送JAK/STAT3通路抑制剂芦可替尼（RUX）。该水凝胶利用RUX的特异性结合位点，实现了高达84.59%±0.73%的药物包封率，并能持续缓释药物超过14天。结合ALG的生物相容性、可降解性以及PNIPAm在体温（37 ℃）下的凝胶化特性，确保了药物的按需释放和在病灶部位的持续作用。体外实验表明，该材料能够对U251和A172胶质母细胞瘤细胞具有剂量依赖性抑制作用。非印迹水凝胶则显示出良好的生物相容性，表明材料本身的安全性。该分子印迹水凝胶为胶质母细胞瘤的术后局部治疗提供了一种安全高效的新策略。

通过在MIPs中引入对特定外界环境刺激或肿瘤微环境中的内部信号（如光照、温度、pH值）敏感的结构单元，可以实现药物的智能控释。其控释机制在于这些刺激能可逆地改变聚合物的物理化学性质（如空腔构象、溶胀度、电荷），从而削弱或破坏印迹空腔与药物分子之间的结合力，最终实现药物在目标部位按需释放。如图5所示，Liu等^［63］^开发了一种pH响应型双功能纳米药物（FASC MIPs），通过双途径阻断雄激素实现协同治疗。该药物以四氧化三铁磁核为基底，表面构建双模板印迹腔，一个特异性捕获肿瘤内睾酮（TETO），另一个负载雄激素受体抑制剂比卡鲁胺（BIC），外层包裹pH响应型壳聚糖（CS）。在酸性肿瘤微环境中，CS外壳质子化降解，触发BIC精准释放并阻断雄激素受体功能，同时印迹空腔能够吸附睾酮，形成双重拮抗机制。体外实验证实，FASC MIPs能有效阻滞LNCaP癌细胞G1期进程，显著抑制其增殖。在荷瘤小鼠模型中，该纳米药物展现出优异的抗肿瘤效果，且未损伤健康组织。

**图5:**
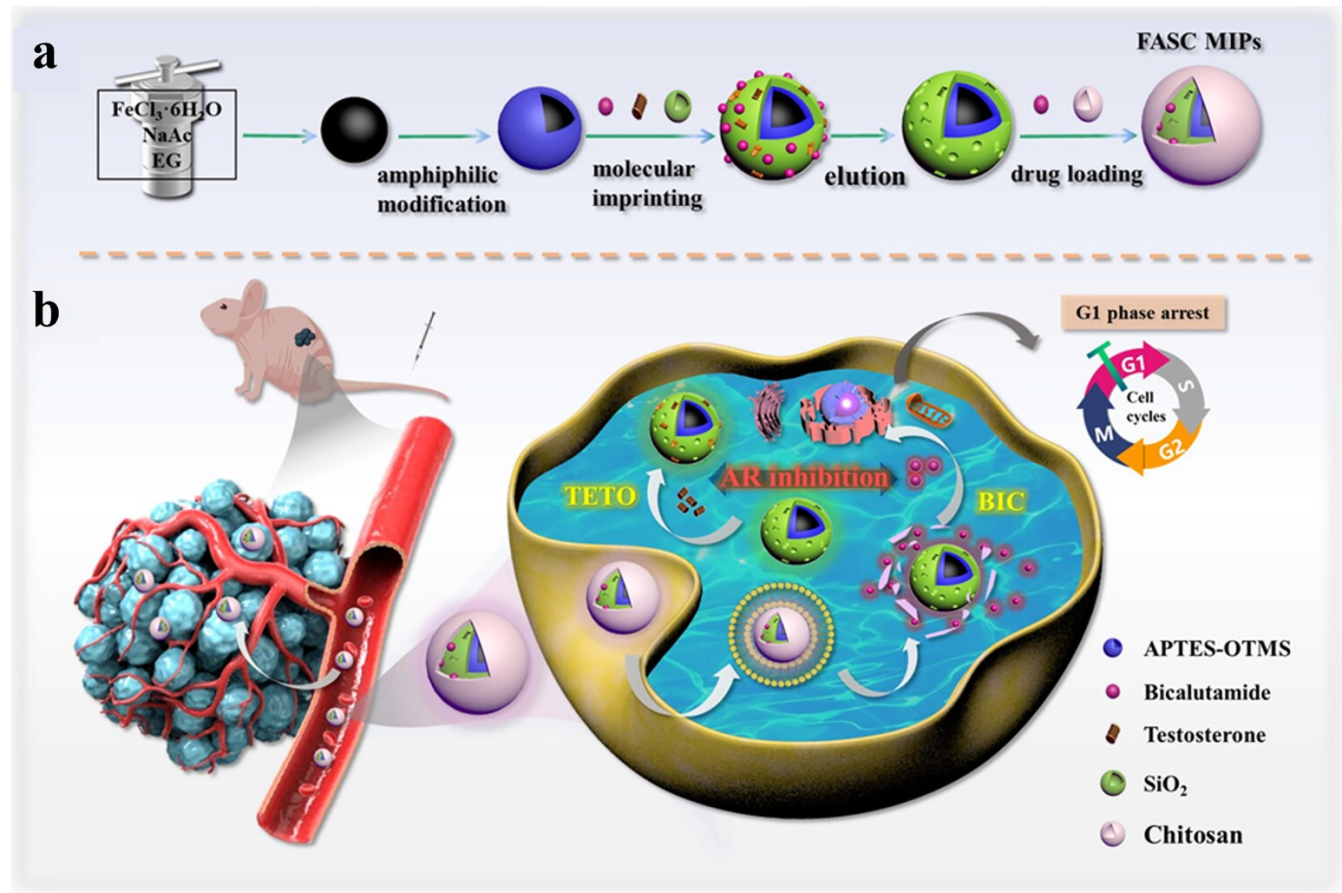
分子印迹聚合物在药物递送中的应用^［63］^

MIPs无需修饰生物配体即可直接利用肿瘤标志物的特异性结合位点实现主动靶向递送，从而显著提高疗效并降低副作用^［10］^。Singla等^［26］^通过固相合成法同步印迹雌激素受体α（ERα）表位并负载化疗药物阿霉素（DOX），从而开发了双印迹MIPs，实现了高效核靶向递药，并具有缓释特性。该材料能特异性结合ERα过表达的乳腺癌细胞，通过受体介导内吞作用将DOX精准递送至细胞核，对ERα阳性细胞系具有显著的细胞毒性，有望成为乳腺癌靶向治疗的低成本解决方案。

#### 2.2.2 光热和光动力疗法

光热治疗（PTT）和光动力治疗（PDT）是两种重要的癌症治疗策略，具有微创性和低毒性的优势。PTT利用光热转换剂吸收特定波长的光（如近红外光）产生热能，选择性升高肿瘤部位温度以诱导癌细胞死亡。PDT则依赖光敏剂在光激发下与组织氧反应，产生活性氧（ROS）而直接杀伤癌细胞。

MIPs凭借其优异的结构可设计性和特异性识别能力，为提升PTT和PDT的疗效提供了强大工具，能够精准靶向癌细胞，显著提高治疗剂在肿瘤部位的富集效率，从而最大限度地降低对正常组织的损伤。例如，Liu等^［64］^利用MIPs的模板识别特性，设计了胶囊状MIPs（DOX@MIP），用于实现靶向、pH响应释药和化学-光热协同的癌症治疗。该研究以多巴胺（DA）作为功能单体、交联剂与光热剂，表皮生长因子受体（EGFR）表位肽作为模板，在载药ZIF-8表面聚合形成印迹层。在印迹过程中，PDA通过螯合作用自发蚀刻ZIF-8内核，最终形成空心胶囊结构（图6a）。该设计通过MIPs的印迹空腔特异性识别EGFR高表达癌细胞；PDA壳层兼具pH响应性及高效光热转化能力，将靶向识别、智能控释、光热治疗集成于单一简易纳米结构，且具有优异生物相容性，为开发低毒高效的多功能肿瘤治疗平台提供了新思路。

**图6:**
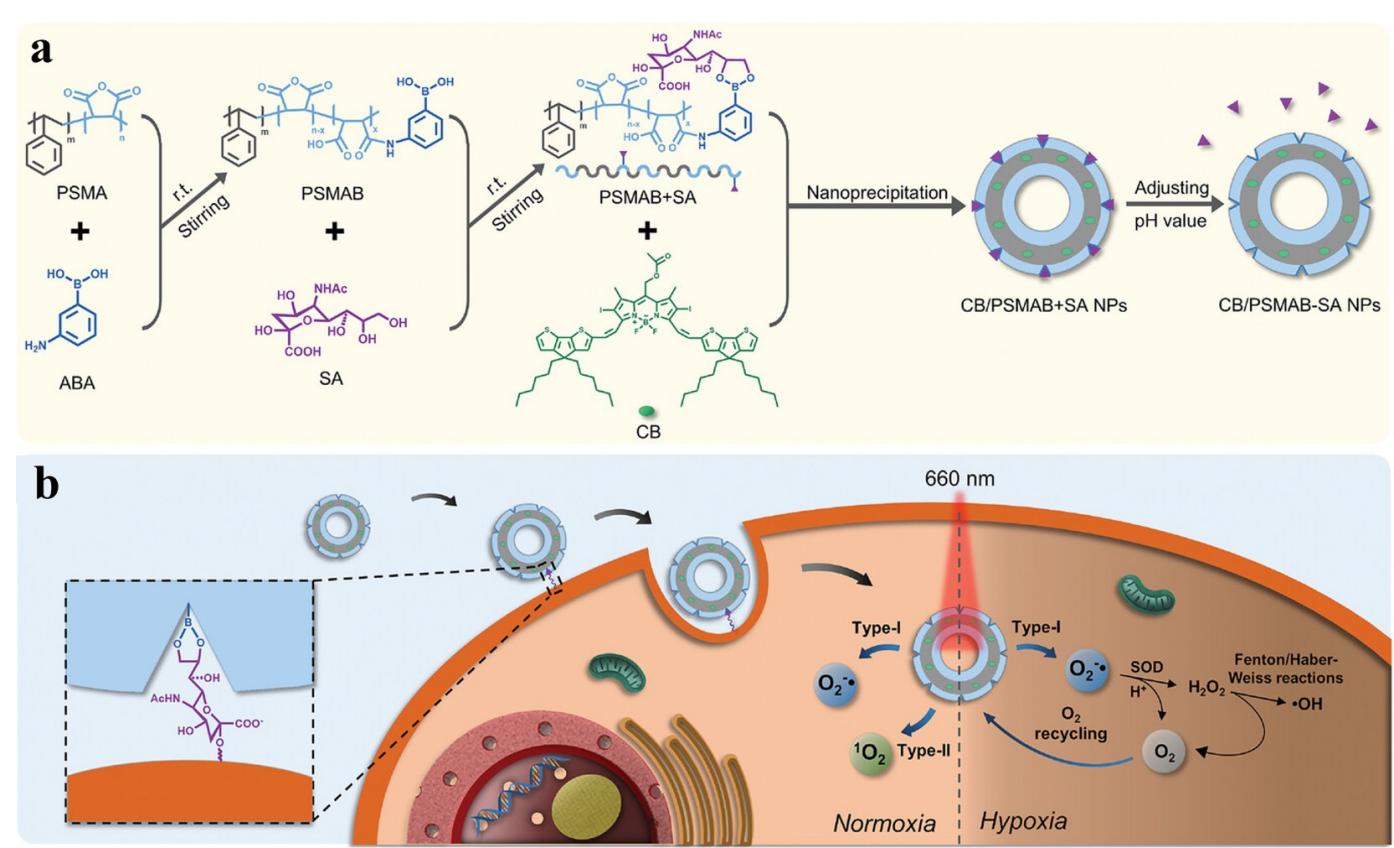
分子印迹聚合物在光热和光动力治疗中的应用^［65］^

为了克服实体瘤中普遍存在的缺氧环境对传统PDT（依赖Ⅱ型机制产生ROS）的限制，Peng等^［65］^以唾液酸（SA）为模板，利用共轭寡聚物与聚苯乙烯-马来酸酐的自组装特性，通过纳米沉淀法制备了一种具有刚性*π-π*堆叠壳层的空心NPs（CB/PSMAB-SA NPs），可选择性地与SA过表达的DU 145癌细胞结合并在癌细胞中积累（图6b）。该纳米体系在660 nm激光触发下展现出独特的ROS生成机制：常氧（20% O_2_）环境下通过Ⅱ型（单线态氧）和Ⅰ型（超氧自由基）双途径产生ROS，而在缺氧（1% O_2_）环境下则优先通过Ⅰ型途径生成超氧自由基，细胞实验证明其在常氧与严重缺氧条件下均能维持显著的光毒性，实现了光敏剂性能调控与肿瘤靶向功能的协同整合。

#### 2.2.3 清除生物毒素

MIT在清除生物毒素方面的研究近年来得到了极大关注，该技术能选择性捕获并清除多种内源性和外源性有害物质，包括农药和蛋白质等^［12，66，67］^。例如，Wang等^［68］^基于近红外持久发光纳米颗粒（LGGO），以农药敌百虫为模板，制备了杂化纳米探针（LGGO@MIP）。该探针在保持91.5%发光强度的同时可特异性吸附敌百虫，并支持LED红光激发的持久低背景体内成像。LGGO@MIP生物相容性良好，口服后分布于胃肠道并随粪便排出，静脉注射后可在血液中循环并最终被肝脾捕获。在敌百虫中毒小鼠模型中，口服LGGO@MIP显著提升存活率至80%并有效缓解症状。

除了针对外源性农药毒素，MIT在内源性有害蛋白（如炎症因子）清除方面也展现出巨大潜力。Bossi等^［16］^制备的纳米陷阱可特异性捕获并清除炎症细胞因子IL-6，降低其在炎症环境中的浓度，从而抑制过度炎症反应并减轻炎症损伤。为了实现对目标蛋白质的彻底清除而不仅仅是吸附捕获，Zhong等^［69］^开发了一种基于分子印迹和酶固定化技术的核壳结构MIP。其外壳可特异性识别结合IL-6，内核则由可降解IL-6的人中性粒细胞弹性蛋白酶/磷酸铜杂化纳米酶构成。体外及小鼠炎症模型实验表明，该聚合物能高效吸附并水解IL-6，显著降低其水平，从而缓解细胞因子释放综合征。通过精妙的设计，MIT已实现对多种有害物质的高选择性和靶向性清除，这些进展为开发新型解毒剂和治疗过度炎症性疾病提供了富有潜力的技术路径。

#### 2.2.4 调控细胞行为

随着现代医学对疾病发生机制的深入解析，MIT通过模拟生物识别原理，能够精确调控生物过程，为疾病治疗提供了靶向干预策略^［12］^。其具体作用机制包括酶活性调控、信号通路干预等^［13，18，67］^。

MIPs可通过特异性结合酶的活性位点，实现空间位阻改变，阻断底物与酶的正常相互作用，从而竞争性地抑制酶活性。Ren等^［43］^构建了一种双模板表位MIPs（D-EMIPs），通过整合靶向识别与核苷酸代谢抑制双重功能实现精准抗癌治疗。该材料同时印迹了人表皮生长因子受体-2（HER2）受体表位肽和肌苷5′-单磷酸脱氢酶（IMPDH）活性中心肽，前者赋予其特异性靶向HER2阳性肿瘤细胞的能力，后者通过占据IMPDH的催化位点阻断鸟嘌呤核苷酸合成，从而抑制DNA及RNA的生成。体外实验表明，D-EMIPs对HER2阳性细胞的增殖抑制率达41%，并且在体内治疗中，对肿瘤的生长表现出明显的抑制效果。Qin等^［70］^提出了一种线粒体靶向的分子印迹聚合物纳米药物（MIP-CTPB），用于抑制肿瘤生长。该研究通过MIT构建了可特异性识别二氢叶酸还原酶（DHFR）活性中心的三维结合空腔，并通过修饰（3-丙羧基）三苯基溴化膦（CTPB）实现线粒体靶向功能。如图7a所示，MIP-CTPB在线粒体富集并与DHFR活性中心结合，通过空间位阻效应阻断DHFR的催化活性，导致DNA合成受阻，达到抑制肿瘤生长的目的。体外实验显示其对HeLa细胞增殖抑制率达42.2%，体内实验显示其显著缩小肿瘤体积至对照组的1/6，且毒副作用较低。

**图7:**
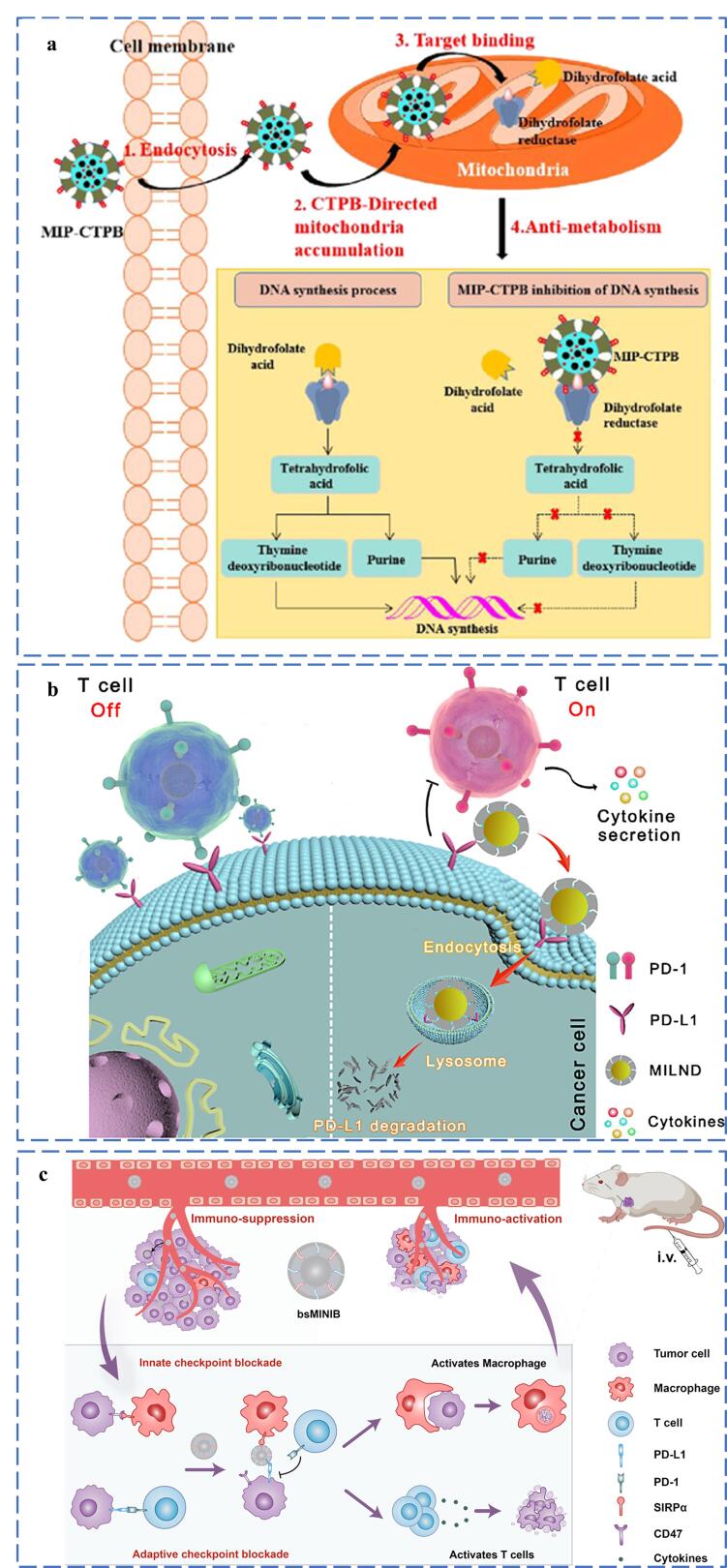
分子印迹聚合物在调控细胞行为中的应用 a. MIP-CTPB^［70］^； b. MILND^［44］^； c. bsMINIB^［45］^.

MIPs还可通过捕获信号分子以阻止它们激活细胞表面的受体，从而干扰信号传导通路，最终实现细胞生命活动的调节。Lv等^［71］^基于缺血性中风的铁死亡等复杂病理机制，设计了一种FeSO_4_模板MIPs。MIPs借助循环中性粒细胞穿透血脑屏障，靶向递送至缺血脑区，有效清除过量Fe^2+^，从而调节多种细胞死亡途径，包括抑制铁死亡、减少细胞凋亡和减轻炎症。Wen等^［72］^开发了hVEGF-MIP通过特异性结合并清除肿瘤微环境中的人血管内皮生长因子（hVEGF），阻断其促血管生成作用，从而抑制肿瘤生长。

此外，免疫检查点阻断也是癌症免疫治疗中关键的信号通路干预策略。Liu课题组^［44］^报道了一种癌细胞靶向分子印迹溶酶体纳米降解剂（MILND）。如图7b所示，该纳米材料通过特异性识别肿瘤细胞PD-L1蛋白的N端表位，促进其内化并转运至溶酶体降解，从而降低PD-L1表达水平，阻断PD-1/PD-L1免疫抑制信号通路，激活T细胞介导的抗肿瘤免疫反应。实验表明，MILND在肿瘤部位高效富集，显著抑制肿瘤生长且无明显毒副作用。为增强抗肿瘤免疫疗效，Liu课题组^［45］^还提出了一种双特异性分子印迹纳米免疫阻滞剂（bsMINIB），选择肿瘤细胞表面PD-L1和巨噬细胞表面SIRPα的特定表位作为模板，如图7c所示，通过同步阻断先天免疫检查点CD47/SIRPα和适应性免疫检查点PD-L1/PD-1信号通路，能够恢复巨噬细胞吞噬功能、促进抗原呈递和激活T细胞杀伤活性。在TNBC模型中，bsMINIB在肿瘤部位的保留时间延长，活性T细胞浸润增强，抗肿瘤巨噬细胞重新激活，从而有效抑制了肿瘤生长。

综上所述，MIT在疾病诊断和疾病治疗领域展现出巨大的应用潜力。表1汇总了MIT在疾病诊断和治疗中的应用，包括制备方法、模板分子、样本类型等。

**表1:** MIT在疾病诊断和治疗领域的应用

Application	Material	Method	Templates	Samples	Ref.
Biosensing	Si-CD/g-CdTe@MIP	surface imprinting	LYZ and Fe^3+^	human serum	［^54^］
	rGO/PDA-MIP	surface imprinting	creatinine， urea， and human serum albumin	human serum and urine	［^55^］
	NSE-imprinted Ag NPs and NSE-imprinted Au NS SAM-coated glass substrate	surface imprinting	NSE epitope	human serum	［^56^］
	AFP/A2G2S2-imprinted Au/AgNP and AFP-imprinted substrate	surface imprinting	AFP epitope and A2G2S2	human serum	［^57^］
Bioimaging	dispersed-phase imprinted nanogel	soild-phase synthesis	PSMA epitope	prostate cancer cells and tissue	［^58^］
	QD-cored cladded MIPs	surface imprinting	HER2 epitope and GPNMB epitope	TNBC cells and TNBC-bearing mice	［^59^］
	Gal-NAc-imprinted nanoparticle	surface imprinting	Gal-NAc	tumor cells	［^60^］
Bioseparation	PP-co-AHH+ hydrogel imprinted with SMMC-7721 cells	cell imprinting	SMMC-7721 cells	human blood	［^42^］
	PS-MIPs	surface imprinting	PS	plasma， urine， amniotic fluid， cerebrospinal fluid， saliva	［^61^］
Drug delivery	MIP_ALG-PNIPAm	bulk imprinting	RUX	Glioblastoma cells	［^62^］
	FASC MIPs	surface imprinting	BIC and TETO	prostate cancer cells and tumor-bearing mice	［^63^］
	ERα epitope and DOX double imprinted nanoMIP	soild-phase synthesis	ERα epitope and DOX	breast cancer cells and 3D cancer models	［^26^］
PTT and PDT	DOX@MIP	surface imprinting	EGFR epitope	cancer cells	［^64^］
	CB/PSMAB-SA NP	nanoprecipitation	SA	prostate carcinoma cells	［^65^］
Biotoxin removal	LGGO@MIP	surface imprinting	trichlorofon	poisoned mice	［^68^］
	GelMA nanotrap	epitope imprinting	IL-6 epitope	human leukemic monocyte THP-1 cell line	［^16^］
	IL-6 imprinted nanozyme	surface imprinting	IL-6	cells and mice	［^69^］
Cell behavior regulation	D-EMIPs	surface imprinting	active center peptide of IMPDH and HER2 epitope	HER2+ tumors cells	［^43^］
	MIP-CTPB	surface imprinting	active center of DHFR	breast cancer cells and tumor-bearing mice	［^70^］
	FeSO_4_ templated molecularly imprinted nanoparticle	bulk imprinting	ferrous sulphate	cells and rats	［^71^］
	hVEGF-MIP	epitope imprinting	hVEGF epitope	breast cancer cells	［^72^］
	MILND	surface imprinting	PD-L1 epitope	4T1 cells and xenograft 4T1 tumor-bearing mice	［^44^］
	bsMINIB	surface imprinting	PD-L1 epitope and SIRPα epitope	TNBC cells and TNBC-bearing mice	［^45^］
	bsMINIB	surface imprinting	PD-L1 epitope and SIRPα epitope	TNBC cells and TNBC-bearing mice	［^45^］

PTT： photothermal therapy； PDT： photodynamic therapy； LYZ： lysozyme； NSE： neuron specific enolase； AFP： alpha fetoprotein； HER2： human epidermal growthfactor receptor 2； GPNMB： glycoprotein non-metastatic protein B； TNBC： triple-negative breast cancer； Gal-NAc： *N*-acetyl-d-galactosamine； PP-co-AHH： poly（polyethylene glycoldimethacrylate-co-acrylamide L-histidine-L-histidine）； PS： phosphatidylserine； RUX： ruxolitinib； BIC： bicalutamide； TETO： testosterone； ERα： estrogen receptor alfa； DOX： doxorubicin； EGFR： epidermal growth factor receptor； SA： sialic acid； IL-6： interleukin-6； IMPDH： inosine 5′-monophosphate dehydrogenase； CTPB： （3-propanecarboxyl）triphenylphosphonium bromide； DHFR： dihydrofolate reductase； hVEGF： human vascular endothelial growth factor； PD-L1： programmed cell death ligand 1.

## 3 结论与展望

近年来，MIT在疾病诊断与治疗领域取得了显著进展，研究者们已成功开发了高灵敏度、高特异性的诊断方法及基于不同机制的靶向治疗策略。然而，该技术在实际应用中面临多种挑战。在制备环节，MIPs对复杂生物分子的选择性仍需提升，尤其是单糖结构的细微差异、蛋白质等生物大分子的构象灵活性及细胞和外泌体等超大模板的流动性、复杂性导致印迹空腔难以精准匹配。同时模板深度嵌入或与空腔结合过强导致去除困难，洗脱过程中易破坏结合位点。此外，复杂样本中的非特异性结合严重制约了识别性能。在应用层面，MIPs用于体内成像或治疗时，需同时满足可降解性、低毒性和强信号的要求，但其生物相容性与长期毒性问题尚未完全解决。加之缺乏规范的临床应用检测标准以及高昂的规模化生产成本，其临床转化存在巨大困难。未来在提升MIPs体内安全性和生物相容性（如采用新型可降解材料、表面亲水性修饰）的同时，还可整合人工智能和大数据技术。通过机器学习预测模板分子与功能单体间的相互作用以优化功能单体组合，筛选最佳合成条件，精准模拟识别过程及评估性能，并结合创新印迹策略和绿色合成方法，系统优化MIPs的制备工艺。这些协同策略将有力推动MIPs在疾病标志物高灵敏检测、即时诊断设备、靶向药物等关键应用场景的实现与应用，加速其从实验室向临床转化的进程。

## 作者团队简介

分子识别与分离分析课题组隶属于吉林大学化学学院。课题组致力于复杂样品高效分离和高灵敏度检测技术的开发，承担了多项国家及省部级科研项目，并与相关领域学者建立了良好的合作关系。

**Figure:**
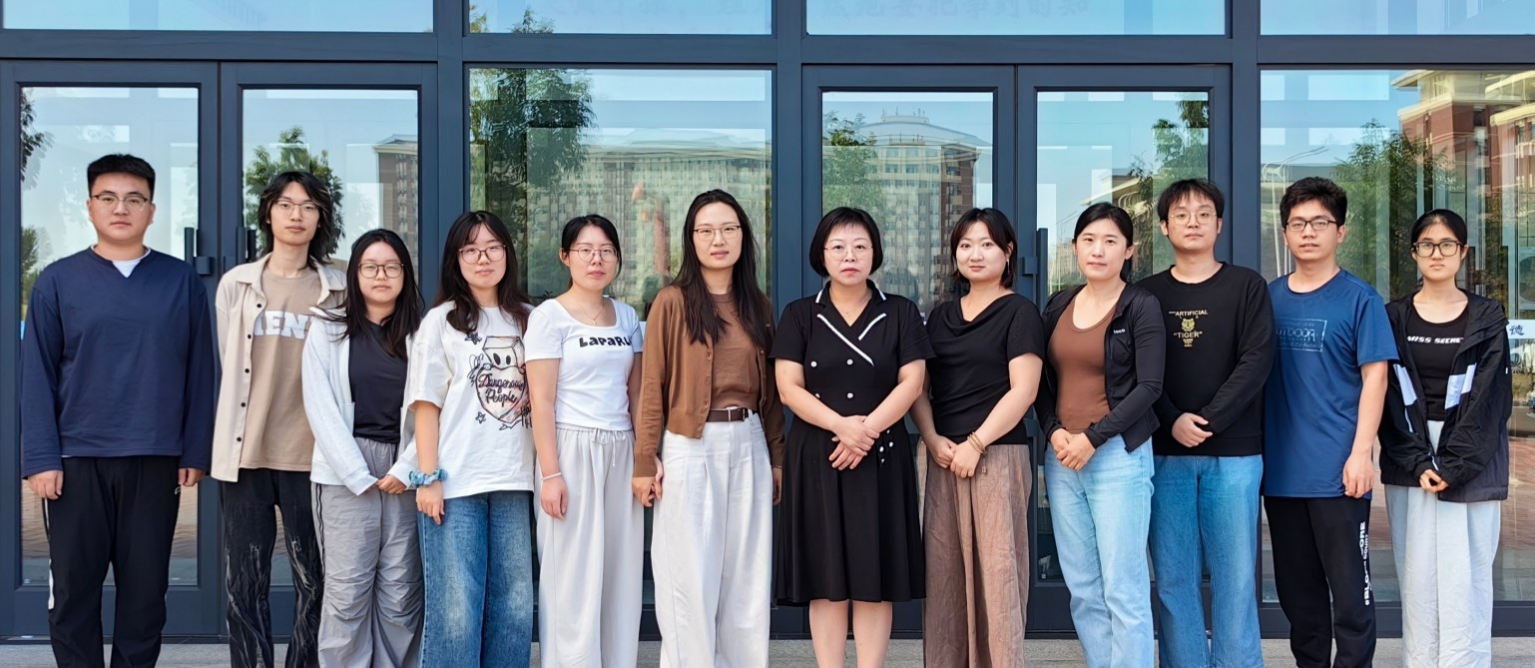


### 人才队伍

**课题组组长：**贾琼教授，吉林大学“唐敖庆学者”。中国化学会高级会员；中国分析测试协会色谱分会、质谱分会、绿色技术分会委员；吉林省土壤污染物检测工程研究中心主任；吉林省分析测试技术学会色谱/质谱专业委员会主任；吉林省人民政府食品安全委员会专家委员会委员；*Chinese Chemical Letters、*《色谱》等期刊编委。

**课题组成员及学生：**教授1人，副教授2人，讲师1人，工程师1人，博士/硕士研究生约20人

**团队精神：**协作共进，励志图强

### 科研项目及成果

**科研项目：**国家自然科学基金；吉林省科技厅、发展改革委、生态环境厅等科研项目

**科研成果：**在*Analytical Chemistry、TrAC-Trends in Analytical Chemistry、Journal of Chromatography A*等学术期刊上发表SCI收录科研论文230余篇，h-因子48，授权发明专利约20件。

**获奖情况：**吉林省自然科学奖二等奖等。
